# A Proposal for a Unifying Set of Definitions of Fatigue

**DOI:** 10.3389/fpsyg.2021.739764

**Published:** 2021-10-13

**Authors:** Simon Skau, Kristoffer Sundberg, Hans-Georg Kuhn

**Affiliations:** ^1^Institute of Neuroscience and Physiology, Sahlgrenska Academy, University of Gothenburg, Gothenburg, Sweden; ^2^Department of Pedagogical, Curricular and Professional Studies, Faculty of Education, University of Gothenburg, Gothenburg, Sweden

**Keywords:** fatigue, definitions, fatigability, effort, sensation of fatigue, performance of fatigue

## Abstract

In this paper, we propose a set of unifying definitions that are useful in all areas of fatigue research while remaining neutral to the various theories about fatigue. We first set up two criteria and four desiderata that a definition for interdisciplinary use needs to fulfill: (i) non-circularity, (ii) finiteness, (iii) broadness, (iv) precision, (v) neutrality, and (vi) phenomenon-focus. We argue that other existing attempts to unify definitions within fatigue research do not fulfill all of these criteria and desiderata. Instead, we argue for a set of stipulative definitions, centered around performance measures and subjective estimations, is required in order to maximize clarity. In total, a set of 13 distinct definitions of fatigue and fatigue-related phenomena is presented. These definitions will help facilitate communication between different researchers, link phenomena from divergent research fields together, facilitate application and knowledge production, and increase the specificity for hypothesis testing.

## Introduction

Fatigue is a phenomenon studied in various research fields, such as cognitive neuroscience, exercise physiology, psychology, and the medical sciences. The sentiment that we need a good and widely accepted set of definitions of fatigue, and related terms, has been echoed by several authors (Hockey, 2013; Kluger et al., 2013; Pattyn et al., 2018). For example, different studies denote the phenomenon of decreased cognitive performance after a period of activity as; central fatigue (Friedman et al., 2007; Kluger et al., 2013), cognitive fatigue (Bailey et al., 2007; Ackerman and Kanfer, 2009; Wylie and Flashman, 2017), mental fatigue (Inzlicht et al., 2014), fatigability (Kluger et al., 2013), cognitive fatigability (Walker et al., 2019), and ego-depletion (Baumeister et al., 2018). The variety of concepts used for the same phenomenon, both within and between different research fields, hinders interdisciplinary collaboration and has the possibility to generate confusion, miscommunication, which affects knowledge production. Additionally, the use of different terms in different fields hinders communication to the extent that advances are kept within one field and do not reach researchers in other fields, leading to the “reinventing-the-wheel” phenomenon. A more ethical problem would occur, if a substantial problem in the research of a phenomenon is detected in one field but not communicated to other fields, since it would expose participants unnecessarily to experiments and potential harms. For example, several multilab studies (Hagger et al., 2016; Dang et al., 2021; Vohs et al., 2021), and several meta-analyses (Carter et al., 2015) have not been able to demonstrate an *ego-depletion* effect in healthy adults, which should be taken into considerations for researchers in other fields that are using similar experimental designs. These problems could be handled once definitions are applied to all areas of fatigue research.

In section “What is Wanted From a Definition to Increase Crossdisciplinarity Communication?,” we propose four desiderata that any definition should fulfill to improve crossdisciplinarity communication. We exemplify how these desiderata can be used to evaluate common definitions in fatigue research. In section “Defining the Performance Part of Fatigue” we define the performance aspect of fatigue. In section “Defining the Subjective Estimation Part of Fatigue” we define subjective estimation of fatigue. From section “Defining the Performance Part of Fatigue” and “Defining the Subjective Estimation Part of Fatigue,” a set of 13 unifying definitions are generated (summarized in Table 1), that are useful in all areas of fatigue research. Technical terms can be found in the Glossary, to guide the reader through the definitions.

**TABLE 1:** The definitions.

Construct	Definition	More lose paraphrases of the definitions
Fatigability (1)	*If and only if* there is the decrement in magnitude or rate of a performance criterion relative to a reference value over a given time of task performance.	*Is the* decrement in performance between two timepoints. *Is the* decrement *in performance over a consecutive time.*
X fatigability (2)	*If and only* if the fatigability of X.	*Is the* decrement *in X performance over a consecutive time.*
Effort (3)	*If an only if* there are forces exerted by the individual in order to reach some goal.	
Sensation of fatigue (4)	*If and only if* there is a sensation of (i) feeling the need for rest, or (ii) mismatch between effort expended and actual performance.	*Is the feeling of either needing to rest or* mismatch between effort expended and actual performance.
Sensation of X fatigue (5)	*If and only if* there is a sensation of (i) feeling the need for X rest, or (ii) mismatch between X effort expended and actual X performance.	*Is the feeling of either the need for X rest or* mismatch between X effort expended and actual X performance.
State fatigue (6)	*If and only if* there is a momentary sensation of fatigue.	*Is the estimation of sensation of fatigue at this moment.*
Trait fatigue (7)	*If and only if* there is an overall disposition and intensity of fatigability and sensation of fatigue, during T period of time.	*Is the general tendency of fatigability and sensation of fatigue.*
Prolonged state fatigue (8)	*If and only if* there is an overall disposition and intensity of fatigability and sensation of fatigue, during the last week.	*Is the general tendency of fatigability and sensation of fatigue, after recent events.*
State X fatigue (9)	*If and only if* there is a momentary sensation of X fatigue.	*Is the estimation of sensation of X fatigue at this moment.*
Trait X fatigue (10)	*If and only if* there is an overall disposition and intensity of X fatigability and sensation of X fatigue, during T period of time.	*Is the general tendency of X fatigability and sensation of X fatigue.*
Prolonged state X fatigue (11)	*If and only if* there is an overall disposition and intensity of X fatigability and sensation of X fatigue, during the last week.	*Is the general tendency of X fatigability and sensation of X fatigue, after recent events.*
Pathological fatigue (12)	*If and only if* the trait fatigue estimated by the individual or caregiver to interferes with usual and desired activities.	*Is when general tendency of fatigability and sensation of fatigue is perceived to interfere with everyday life.*
Pathological X fatigue (13)	If and only if the pathological fatigue identifiable as caused by, or consequence of, or sequel to a disease/disorder/trauma and *if and only if* the level of trait fatigue is worse after the disease/disorder/trauma than before.	*Is when the general tendency of fatigability and sensation of fatigue is perceived to interfere with everyday life and is caused by, or consequence of, or sequel of X.*

## What Is Wanted From a Definition to Increase Crossdisciplinarity Communication?

To increase crossdisciplinarity communication, we need some form of consensus about the central terms used to describe the phenomena the field aims to explore. A disagreement about a phenomenon, e.g., fatigue, is interesting, and could potentially lead to studies advancing the field, but a disagreement that is just the result of attaching different meanings to the same terms, a mere verbal dispute, is neither interesting nor productive. For us to have interesting *dis*agreements about fatigue, mental fatigue, etc., we must first have an agreement about the meaning of the terms. We have to agree to disagree, as it were. For that, we need definitions. The most common definitions in science are real and stipulative definitions. Importantly, what counts as a successful or unsuccessful definition is different for real definitions and stipulative definition. A real definition could be right or wrong (e.g., it could be wrong because it is contradicted by empirical evidence), but a stipulative definition is useful or not useful (e.g., it could be circular, or too narrow/broad/vague to be useful for a particular purpose). Real definitions are when we relate a *species*, the basic/smaller units of classification, to a *genus*, the higher order/group unit of classification. For example, male and female are two different species of the genus adult human beings. A real definition aims at finding the real or essential characteristics of the thing or phenomenon in question. To discover the real definition of a term one needs to investigate the thing or things denoted by the term. However, useful this might be in other scientific enterprises, in the field of fatigue we would argue, the classifying units are at the moment too vague. Thus, to increase crossdisciplinarity communication, we would argue the need right now is rather to find a way to reach consensus about the terms used, meaning that we need a set of stipulative definitions. A stipulative definition is the introduction of a new term:

“For instance, the sentence ‘Someone has ARDS if and only if she has an acute respiratory distress syndrome’ is a definition of the new term ‘ARDS.’ It introduces this term as a short name for the longer sequence ‘acute respiratory distress syndrome’ and establishes the syntax of its use (‘x has ARDS’) in order that one avoids to say, for example, ‘x bears ARDS.’ Thus, a [stipulative] definition is always a nominal definition (nomen = name), and as such, it is a stipulative sentence that introduces a term, and is never a constative or descriptive sentence to state or assert something. For instance, the term “ARDS” describes or reports nothing. Definitions are uninformative. They are only regulative and useful.” (Sadegh-Zadeh, 2015, p. 95).

Technically, a definition^1^ such as “X is Y,” is made up of the *definiendum*, the word or phrase defined in a definition (in this case X), and the *definiens* (plural *definientia*), the sentence or phrase that defines the definiendum (in this case Y). The goal of this paper is to suggest novel and better definitions of key terms. In other words, we will offer stipulative definitions of key terms in the manner of: “Fatigue *is* X” or “X *is* Y and Z.” But what does “is” mean in a stipulative definition? When we say “X *is* Y,” we mean that everything that is true of X is also true of Y, and everything that is false of X is also false of Y. The “is” in a stipulative definition can thus be translated to “if and only if” (called a *biconditional*) e.g., “X *if and only if* Y.”

There are two minimal criteria that any stipulative definition needs to fulfill in order to be a successful definition (see chapter 6 in Sadegh-Zadeh (2015) for further discussion on definitions):

i) *Non-circularity*, i.e., no part of the term defined (definiendum) should be defined by itself or have already been used in the definitions of a prior definition.
ii) *Finiteness:* the definition chain cannot be infinite, i.e., at the end of our definitions, there should be some *primitives* or undefined terms that typically get their meaning from *ostensive procedures*.

For example, if fatigue was defined as “the feeling of exhaustion, weariness or lack of energy,” and exhaustion in turn was defined by “the feeling of fatigue, weariness or lack of energy,” then fatigue would both be the definiendum and the definiens, making it circular. The purpose of the *finiteness* criterion is that the definition needs in some way be related back to reality. Thus, it might be that X is defined by Y, and Y is defined by Z, but at the end of the definition chain Z needs to be defined by something like Q, where Q is an undefined primitive which gets its meaning from meta-linguistic practices, such as an ostensive procedure, e.g., “This (pointing to Q) is Q,” “the color marine blue is this (pointing to an object that is marine blue)” or “what you are feeling now (indicating this moment) is angst.” In our natural language, words and terms do not always fulfill these two criteria, but regardless of how hard it is in our scientific language they need to be fulfilled or the terms lose their connection to reality.

However, fulfilling only these minimal criteria will not be sufficient to serve as candidates for useful and clear definitions across research fields. In addition, we propose four *desiderata* i.e., something that we want or desire from a definition to be considered a good definition in terms of it being applicable across research fields.

iii) Broadness: the definitions are broad enough to be used in a variety of research fields.
iv) Precision: the definitions are precise enough to avoid multiple interpretations.
v) Neutrality: the definitions should not appeal or depend on any particular theory.
vi) Phenomenon-focus: our definitions to a minimal extent involve explanations, since our goal is to reach consensus about the phenomenon explained (explanandum) and not about the explanations (explanans).

We will show how these criteria and desiderata can be successfully fulfilled. We will use an example of a diagnosis, and though this paper is not aiming to provide any diagnosis of fatigue, it is a useful example of how the regulation of a stipulative definition work. “Agoraphobia is characterized by marked and excessive fear or anxiety that occurs in response to multiple situations where escape might be difficult or help might not be available” (WHO, 2021). This fulfills the *non-circular* and *finiteness* criteria since none of the terms “excessive fear,” “excessive anxiety,” “escape,” “help” is defined by “agoraphobia” and the definition chain ends with primitives or diagnostic criteria’s. It fulfills the *broadness* desideratum since it is applicable and used in the same way in various fields. It fulfills the *precision* desideratum since it gives criteria for what it means for someone to have agoraphobia. It does not depend on any specific theory of fear or anxiety and thus fulfills the *neutrality* desideratum. Lastly it fulfills *phenomenon-focus*, since the “occurs in response to,” is not an explanation but rather part of the state of affair and thus a phenomenon.

Now that we have established how the criteria and the desiderata work, we analyze two definitions of fatigue used in the literature. Although both fail to fulfill all four desiderata, they both are successful in highlighting important properties or structures needed to be taken into account in any general definition of fatigue.

“[Fatigue is] a subjective lack of physical and/or mental energy [and] which is perceived by the individual or caregiver to interfere with usual and desired activities.” (published by The Council for Clinical Practice Guidelines and the Paralyzed Veterans of America in 1998 cited in Béthoux (2006).

We can begin our analysis by defining “subjective lack of,” “mental energy,” “physical energy,” “perceive to interfere,” “caretaker,” and “usual and desired activities.” If none of these terms and their defining phrases refer back to any of the other, they fulfill the *non-circular* criterion, e.g., defining “mental energy” should not involve the terms “subjective lack of” or “fatigue.” If we suppose the continuation of the defining process ends with a satisfying primitive, then the *finiteness* criterion is fulfilled. In this case, a possible end to the definition chain could be “mental energy is this or that (pointing out a behavior, a feeling or an experience)” or “a caregiver is such and such.” It might be jarring to say that “mental energy” is a primitive, but for the sake of argument we suppose that it works, and we will shortly show that the concept is problematic for other reasons. The definition does fulfill the criterion of *phenomenon-focus*, since no part of the definition invokes an explanation.

This definition of fatigue is used within the medical science, but does it fulfill the desideratum of *broadness*? Both yes and no. As a specific definition of what we later will call subjective estimations of fatigue, it could be used in various field from exercise science to medicine. As an overarching definition aiming to encapsulate all aspects of fatigue, however, it would not meet the *broadness* desideratum, since there are some parts of the study of fatigue that are not captured. Typical study designs in other fields would not fulfill this definition. For example, an exercise scientist aiming to study the effects of intense training on cognition or a social psychologist wanting to study the effect of sustained attention on cognition, both set up their experiments in a way that the participants either do prolonged period (e.g., 1 h) of intense training or a sustained attention task followed by a cognitive task. However, the participants respond to the training or the sustained attention task, if the activity is not “perceived as interfering with usual and desired activities,” such as continuing working or studying after the experiment, then this would not be fatigue according to the definition. As such the definition is too narrow.

Also, the invocation of mental energy creates a problem. Fatigue has often been described with the help of the metaphors such as “lack of energy” or “running on fumes.” A metaphor “is understanding and experiencing one kind of thing in terms of another” (Lakoff and Johnson, 2003, p. 5), to reveal or create structural similarities. A metaphor follows the formula of “X is *like* Y,” which is different from “X *is* Y.” In some cases, it can create a false equivalence of the two, leading to mistaking the map for the world, as it were. However, intuitive a metaphor might be, it is vague by nature and should be avoided as part of a stipulative definition for the sake of clarity (Hockey, 2013; Pattyn et al., 2018), and as such it fails the *precision* desideratum. On the other hand, if mental energy is not used as a metaphor, it could be understood as a theoretical construct. If this is the case then it should not be interpreted or used differently between theories, otherwise it can seriously confuse the debate. For example, are we referring with “mental energy” to Spearman’s theory of intelligence (Spearman, 1927) or O’Connor’s three dimensional model (O’Connor, 2006) or something else? Thus, by using mental energy as a theoretical construct, it will fail the *neutrality* desideratum.

A commonly used and promising candidate for being a unifying definition of fatigue is one given by Aaronson et al. (1999). They set out to propose a definition of fatigue based on their research:

“[Fatigue is] The awareness of a decreased capacity for physical and/or mental activity due to an imbalance in the availability, utilization, and/or restoration of resources needed to perform activity (…) Fatigue occurs when this system is out of balance – that is, when there are insufficient resources either because the demand or need is too great or because mechanisms of utilization and restoration are disturbed.” (Aaronson et al., 1999, p. 46).

This definition of fatigue is made up of two propositions and a connective that links the two propositions. (A) “the awareness of a decreased capacity,” (B) “imbalance in the availability, utilization, and/or restoration of resources needed to perform activity,” and (C) the connective “due to,” that links (A) and (B) together. We will now examine how each part fails to fulfill at least one of our desiderata for consensus use, and as a consequence either leads to confusion, misunderstanding, or a likelihood not to interpret it literally.

The problem with (A) is that the use of “awareness” has several disadvantages which make it fail to fulfill both the *broadness* and *precision* desiderata. Firstly, awareness is too cognitive and enables an interpretation such as “Diana abstractly theorizes that she has a decreased capacity.” The fact that this interpretation is possible, together with other possibilities like “Diana *feels* that she has a decreased capacity,” makes it too broad and thus fails the *precision* desideratum. Secondly, “awareness” is a success term. Just like seeing is a success term to the extent that when our visual perception does not match reality, we call it illusion or hallucination, rather than a state of seeing. The same is true for awareness, i.e., if Diana is aware of X, then X is the case. This on the other hand makes it too precise, and thus fails the *broadness* desideratum, since in many fields of study, such as exercise science and medical science, one is interested in studying situation where individuals have the feeling of not being able to continue with an activity, without it actually being the case that they cannot continue with the activity.

The problem with (B) is that it fails the desideratum of *phenomenon-focus*, since it is an explanation not required for identifying the phenomenon. The problem with (C) is that “due to” is too strong, and fails the *broadness* desideratum. A literal interpretation of this definition requires Diana to have the awareness of the cause of the imbalance. In some circumstance one might know the cause, like after a long day at work, but there are other situations, perhaps due to an undetected tumor or hormonal imbalance it is not known and would imply that a patient seeking help for fatigue is not fatigued according to this definition, and thus this definition would not be useful when studying patients suffering from fatigue or fatigue related problems within the medical sciences. There are, however, many good parts to the definition, which we will come back to later toward the end of section “Defining the Subjective Estimation Part of Fatigue”, but we first need to define some related terms.

In a paper aiming to put forward a unifying taxonomy for fatigue, Kluger et al. (2013) argue that when dealing with fatigue, we should distinguish between the “subjective sensations” and the “objective changes in performance.” We will make a similar distinction between the phenomenon identified by “subjective estimations,” by Kluger et al. (2013) called perceptions of fatigue, and the phenomenon identified by “objective measurements,” which denote the performance of fatigue. Section “Defining the Performance Part of Fatigue” deals with the performance of fatigue, and section “Defining the Subjective Estimation Part of Fatigue” with the subjective estimations of fatigue.

## Defining the Performance Part of Fatigue

In this section, we will start out with suggesting novel or improved definitions of phenomena related to the performance part of fatigue. We will anchor our positive account in already established definitions from the literature and see if they meet our desiderata. In this section, definition (1 and 2) will be presented. Kluger et al. (2013) discuss the concept of fatigability at the performance level of fatigue:

“fatigability is defined as the magnitude or rate of change in a performance criterion relative to a reference value over a given time of task performance or measure of mechanical output” (Kluger et al., 2013, p. 411).

This definition fulfills most of our desiderata except for *precision*, since it does not specify in which direction the change needs to go in order for it to count as fatigability. The last part, “measure of mechanical output,” is made redundant by the “relative to a reference value over a given time.” We would argue that the “over a given time” would suffice. With these few alterations, we suggest the following definition.

(1) Fatigability is the decrement in magnitude or rate of change in a performance criterion relative to a reference value over a given time of task performance.

It is important to highlight here that the definition of fatigability excluded the possibility of something being fatigability when there is no performance change or lack of improvement. For example, in Skau et al. (2019), we found that patients suffering from problems with fatigue after a mild traumatic brain injury (TBI) performed on a cognitive task (Digit Symbol Coding) (Wechsler, 2010) equivalently well at two time points that were intermediated with 1.5 h of intense cognitive activity. At the same time, healthy controls improved their performance. According to definition (1), this would not be fatigability in the TBI patients. Of course, one could change the definition to involve a healthy population reference group. By not improving like they possibly would, one could call it fatigability in relation to some fatigability quotient. Although desirable, we would argue that it is not needed to identify the phenomenon in question and is not needed for our purposes. One could also define another term, let us say “improvability,” and claim that they fail to fulfill that criterion. That is also a desirable thing to do, but again, it is not needed to talk about fatigability. In the same study, the TBI patients also rated their state fatigue (more on this later) before and after the cognitive activity. That they reported being more fatigued after the experiment compared to before does not mean fatigability since the task of reporting on one’s subjective state is not a performance but rather an estimation.

What type of fatigability a researcher is interested in varies, be it physical, mental, cognitive or emotional fatigability. Whether cognitive or emotional fatigability exist depends on what is involved in the term “cognitive” or “emotional,” and is part of the theories of the different research domains. With definition (1) we can add the domain of inquiry/the domain affected to the definition, e.g., “X fatigability,” where X can be replaced by different domains such as “*cognitive* fatigability” or “*physical* fatigability.” This would help communication between different research field and generate transparency. For example, from definition (1), we can derive a fatigability effect, i.e., the difference in performance between time point t_1_ and time point t_2_, and the larger difference, the more fatigability. In social psychology, the focus has been on the ego-depletion effect, which is identical to the fatigability effect. It is only the theories [such as the strength model (Baumeister et al., 2018) and motivational theory (Inzlicht et al., 2014)] and explanatory constructs of willpower and self-control that are different from other fields such as medicine. For example, in both social psychology (Carter et al., 2015), exercise science (Yanagisawa et al., 2010; Alves et al., 2012), and medical sciences (Skau et al., 2019) the unit of measurement are change in reaction time on a Stroop task, and could thus be denoted “cognitive fatigability,” since the Stroop task is a classic cognitive task. Thus, we propose the following definition:

(2) X fatigability is the fatigability of X.

An alternative for researchers that would still want to keep their “within-research field terminology,” such as “ego-depletion,” is that the applicable definition is adapted and integrated, e.g., one could write the following: “Our results show an ego-depletion effect (the within research field terminology), in other words a cognitive fatigability effect (the cross-research field terminology).” The same holds for phenomena such as physical fatigue and motor fatigue. As definition (2) is formulated, only the domain affected (X) is determined. If one wants, it is possible to add “where the effort is of Y” e.g., cognitive fatigability where the effort is of physical/mental/cognitive/emotional performance or something else.

A term often related to performances is that of peripheral fatigue. Torres-Harding and Jason define Peripheral fatigue as: “failure to sustain force or power output because of ‘failure in neuromuscular transmission, sarcolemmal excitation, or excitation-contraction coupling,’ implying neuromuscular dysfunction outside of the central nervous system, or CNS” (Torres-Harding and Jason, 2005). This is how many definitions of peripheral fatigue are constructed (Wylie and Flashman, 2017), however, if we take out the explanatory part, we end up with “failure to sustain force or power output.” This failure would be equivalent to *fatigability* or *physical fatigability*, which is why we do not include peripheral fatigue in our set of definitions. The same argument goes for the term central fatigue. We do not advise against the use of central and peripheral fatigue, since it is part of many taxonomies and is used relatively consistent in the literature, but we want to point out that it often serves as an explanation of mechanisms behind a phenomenon, and we are here only interested in consensus about the phenomenon explained.

## Defining the Subjective Estimation Part of Fatigue

In this section we will discuss the definitions of the subjective estimation part of fatigue. Here definition (3–13) will be presented in a consecutive order. As in the previous section, we will begin our discussion with the work of Kluger et al. (2013), who use the term “perceptions of fatigue” as follows:

“Perceptions of fatigue refer to subjective sensations of weariness, increasing sense of effort, mismatch between effort expended and actual performance, or exhaustion (Kluger et al., 2013, p. 411)”.

Here, the authors define perception as a subjective sensation, but we propose to only denote it “sensation of,” for the sake of brevity^2^. The above definition is a disjunctive made up of four parts “weariness,” “increasing sense of effort,” “mismatch between effort expended and actual performance,” and “exhaustion.” We will first discuss “increasing sense of effort” and “mismatch between effort expended and actual performance,” since it introduces the term “effort.” Kluger et al. (2013) do not define effort, but Massin (2017) showed in an overview of different accounts of effort (i.e., theories of effort), that both the resource-based accounts and the force-based accounts are functionally equivalent, but that force-based accounts are explanatorily more fundamental. Even though our desideratum of *neutrality* implies that we should avoid definitions of terms that depend on a particular theory, since the force-based account and the resource-based accounts are functionally equivalent, this definition will be as broad as possible. Thus, we will use the force-based account statement of effort.

(3) (Effort is) the forces exerted (by the individual) in order to reach some goal (Massin, 2017, p. 243).

Since the goal is the individual’s goal, “the force exerted” needs to be part of the individual’s volition, i.e., that applying the force to some extent is optional. The optionally applied force is aimed to meet the demands, which the individual perceives, to be required to reach the goal. Here the word “perceives” is used in its broadest form, in a way that a mouse perceives what to do when facing an obstacle. Although there is a close relationship between effort and fatigue, having sensation of “increasing sense of effort” with our definition (3) would mean that every time there is a sensation of increasing sense of “force exerted,” there would be a sensation of fatigue, which would be too broad to be useful. Instead, we propose eliminating “increased sense of effort” but keeping “mismatch between effort and actual performance.”

Regarding the concepts of weariness and exhaustion, we need to be careful not to break the criterion of *non-circularity*, as illustrated in section “What is Wanted From a Definition to Increase Crossdisciplinarity Communication?”. Choosing to define fatigue in terms of weariness and exhaustion sets certain strict limits on how they can be defined. While it is tempting to define fatigue in terms of weariness and/or exhaustion, and similarly tempting to define exhaustion and/or weariness in terms of fatigue, we must choose one or the other to avoid circularity. Given these considerations, how do Kluger et al. (2013) define weariness and exhaustion? Unfortunately, neither weariness nor exhaustion is expanded upon by them so we cannot know what exact definition they had in mind. Possibly, they had no specific definitions in mind, but instead wanted the terms to be treated either as primitives or as undefined terms in order to be defined in the future or by others. While, as was just argued, we *could* define fatigue in terms of weariness and/or exhaustion, it is still an open question whether we *ought* to do so.

When it comes to mechanistic explanations or definitions of performance, there are cases where fatigue and exhaustion are defined differently (Aaronson et al., 1999). Thus, these would be available as means of defining fatigue without breaking the criterion of *non-circularity*. Unfortunately, in the definition of the sensation of fatigue it is specifically *the sensation* of exhaustion and weariness that is part of the definition. When it comes to the sensation of fatigue, exhaustion and weariness, they are commonly used as synonyms (Kristensen et al., 2005; Loy et al., 2018; Boolani et al., 2019), which would reintroduce the circularity. Even so, attempts to define the sensation of fatigue do point at two other phenomena that are not used as synonymous of fatigue and which might serve better in terms of fulfilling the four desiderata: *exertion* and *tiredness*.

One such attempt is Phillips’ review of definitions of fatigue. He highlights that any whole definition of fatigue needs to take into account the experience of fatigue (which is what this section is about) and he also highlights the importance of exertion and tiredness:

”However, popular use of the word in everyday language in phrases like “mental fatigue,” “adrenal fatigue” or “battle fatigue” do seem to reflect dictionary definitions in that someone or something is “tired” to the extreme specifically because of some overuse, overexposure or exertion. Capturing this would thus seem to be important for the face validity of a whole definition of fatigue (…) A whole definition would do well to maintain face validity by describing how fatigue is experienced as a result of exertion” (Phillips, 2015, p. 49 and 53).

Let us consider exertion and tiredness. As we have defined effort previously, it seems exertion cannot be understood as an independent term [for a good discussion of effort and exertion see Steele (2021)]. Exertion is accounted for by definition (3) of effort. If we expand the sensation of “mismatch between effort and actual performance” with our definition (3) it becomes “mismatch between ‘the forces exerted by the individual in order to reach some goal’ and actual performance.” Thus, unfortunately, exertion does not add anything that is not already accounted for by our definition of effort.

Tiredness, on the other hand, is not accounted for by our definition of effort. In Phillips’ review it is made clear that although the experience of tiredness is part of the experience of fatigue, tiredness should not necessitate sleepiness (Phillips, 2015), i.e., it should not be the case that every time Diana feels sleepy, she also feels fatigue, violating the *precision* desideratum. But what does “tiredness that does not necessitate sleepiness” mean? We again suggest that we leave the term tiredness behind and instead use “feeling the need for rest.” The “need for rest” is both broad enough to include sensations of participants in exercise studies, as well as the reports from stroke patients. It is also precise enough since “need for rest” does not necessitate sleepiness. With these modifications, we propose the following definition:

(4) Sensation of fatigue is the sensation of (i) feeling the need for rest or (ii) mismatch between effort expended and actual performance.

We can now generate a further construct by adding a dimension/domain as we did in the previous section.

(5) Sensation of X fatigue is the sensation of (i) feeling the need for X rest, or (ii) mismatch between X effort expended and actual X performance.

Here X can be cognitive/mental/physical/emotional or whichever domain the research in question is about.

An additional important distinction to make is that between state and trait. State and trait are commonly used with constructs such as anxiety and fatigue. State usually refers to how an individual “feels here and now,” whereas trait denotes something more latent that does not quickly change. An inconvenience with this terminology, is that state, in contrast to a process and event, is sometimes defined as something having homogenous temporal parts (Mulligan and Smith, 1986), thus the difference between state and trait cannot be reduced to having heterogenous or homogenous temporal parts. Thus, we need in our definition make sure that the difference is due to the transitory aspect of the state, and the long-lasting property of trait (Julian, 2011). Thus, we will keep this terminology due to its broad and consistent use, but in the definition make the time span more explicit.

(6) State fatigue is the momentary sensation of fatigue.

That state fatigue is momentary means that it can change relatively fast within minutes or hours as other sensations can. Definition (6) has a peculiar property that needs to be highlighted. If the condition of the sensation of fatigue is not fulfilled, then there is no state fatigue according to this definition, i.e., if Diana does not have any sensation of feeling the need for rest, or a mismatch between effort expended and actual performance, at this moment, then we cannot say that she has any state fatigue at all, but rather a lack of state fatigue.

On the other hand, trait fatigue is more stable and enduring and does not change rapidly but over weeks, months, or years.

(7) Trait fatigue is the overall disposition and intensity of fatigability and sensation of fatigue, during T period of time.

Since trait fatigue is defined as a disposition, it means that every human always has trait fatigue to a varying degree since a disposition to never have fatigability or sensation of fatigue is still a disposition. That the time clause (T period of time) is, to some extent, arbitrary. It could just as well be 3 weeks or few months or years. This period of time should best be fixed by the researchers within the different fields. Even if T was 3 weeks or years the same phenomenon is denoted, it is only different practices between the fields that are different. However, the time should not be much shorter since there is an intermediate phenomenon that is more stretched out in time than state fatigue but does not have the same characteristic of trait fatigue. This intermediate phenomenon is often recognized when trying to estimate trait moods. One does not want something unexpected that just happened recently, within a few days, to affect the estimation. We will denote this “prolonged state fatigue.”

(8) Prolonged state fatigue is the overall disposition and intensity of fatigability and sensation of fatigue, during the last week.

The difference between the phenomenon of trait fatigue and prolonged state fatigue is the effect of recovery. Let us say that Diana has relatively low trait fatigue, but due to intense stress and lack of sleep during the workweek, her performance on Friday gets quickly worse, and she has an intense sensation of fatigue. However, one day of rest and a good night’s sleep would change that. This would not be the case for trait fatigue, where only one night of sleep would not automatically change her dispositions.

All these definitions (6–8) can be extended to:

(9) State X fatigue is the momentary sensation of X fatigue.
(10) Trait X fatigue is the overall disposition and intensity of X fatigability and sensation of X fatigue, during T period of time.
(11) Prolonged state X fatigue is the overall disposition and intensity of X fatigability and sensation of X fatigue, during the last week.

Here X refers to a specific domain such as cognitive/mental/physical/emotional, whereas T is a period of time. The final definition is that of pathological fatigue. There are several diagnoses related to fatigue that require the cause of the fatigue to be identifiable, e.g., for cancer-related fatigue, the fatigue needs to be caused by cancer (Mitchell, 2010), for exhaustion disorder, the fatigue needs to be caused by a prolonged stressful work period or environment (Jonsdottir et al., 2013). We propose to divide it into two separate definitions.

(12) Pathological fatigue is the trait fatigue estimated by the individual or caregiver to interfere with usual and reasonable desired activities.
(13) Pathological X fatigue is the pathological fatigue identifiable as caused by, or consequence of, or sequel to a disease/disorder/trauma and if and only if the level of trait fatigue is worse after the disease/disorder/trauma than before.

Definition (12), similar to that of Béthoux (2006) (which was analyzed in section “What is Wanted From a Definition to Increase Crossdisciplinarity Communication?”), does not relate pathological fatigue to any source, whereas (13) does. The addition of “reasonable” desired activities should here be understood as activities that are within the realm of possibilities for the person to do, i.e., if Diana perceives her trait mental fatigue to interfere with her desire to run a marathon every day, that would not be reasonable in this sense and hence not pathological fatigue, whereas interference with spending time with friends and family or work would.

Definition (13) is not meant to exclude or replace any diagnoses. On the contrary, it is instead meant to relate the different diagnoses to the other definitions, e.g., cancer-related fatigue is pathological cancer fatigue, or exhaustion disorder is pathological stress-related fatigue. The biconditional within the definition (13) is added to enable the use of the broader and vaguer terms “the consequence of” or “sequel to.” Otherwise, the definition would get the embarrassing property that even if an individual got a lower degree of trait fatigue after the disease/disorder/trauma that would be seen as pathological cancer fatigue. Definition (13) leaves it open for each fatigue-related problem to have additional symptoms or signs. For example, a sensitivity to light and sound is common for individuals suffering from fatigue after exhaustion disorder or TBI (Johansson and Rönnbäck, 2014), which is not part of definitions (1–13).

Now that all definitions are done one could try to generate a general definition of fatigue as we discussed in the end of section “What is Wanted From a Definition to Increase Crossdisciplinarity Communication?”. If we change awareness to a sensation, then it is possible that Diana can have the sensation X, while X not being the case, e.g., Diana can have the sensation as of decreased capacity for physical activity, without the actual presence of a decreased capacity for physical activity. This would fulfill both the *broadness* and *precision* desiderata. A proposal would be that fatigue is “the presence of fatigability or the sensation of fatigue.” Although this definition would solve the problems presented in section “What is Wanted From a Definition to Increase Crossdisciplinarity Communication?” (e.g., the disjunct would solve the “due to” problem), we would argue that such a definition will not be useful. There is still an open question about to what extent fatigability and sensation of fatigue are related, and as phenomena they are separate and indeed many times studied separately. Having the term “fatigue” defined as such could generate confusion since it would not be clear whether one studied fatigability, sensation of fatigue or both. To refer to fatigue in this way, as a disjunct, might be useful in everyday language, but we suggest that it ought to be avoided in scientific discourse. All definitions^3^ are summarized in Table 1, together with possible paraphrases that keep the meaning of the more precise definitions.

## Conclusion

The proposed set of unified minimally theoretical definitions are summarized in Table 1. These constructs can now be imputed with empirical data. Taxonomies can be created or related to the definitions from different fields and theories, that can help settle both verbal or a genuine difference between studies/theories/research fields. It can be applied when comparing the over 250 different scales created to measure fatigue (Hjollund et al., 2007). The definitions are created to the effect that constructs such as emotional fatigue, physical fatigue, or stress fatigue, as other researchers has investigated, can be applied, for instance to definitions (2, 5, 8–10). The definitions (2, 5, 8–10) are also formulated in such a way that such definitions might be redundant, and most importantly, all definiens are usable in all research fields of fatigue.

## Data Availability Statement

The original contributions presented in the study are included in the article/supplementary material, further inquiries can be directed to the corresponding author/s.

## Author Contributions

SS: conceptualization and writing first draft. KS: writing – review and editing. H-GK: writing – review and editing. All authors contributed to the article and approved the submitted version.

## Conflict of Interest

The authors declare thats the research was conducted in the absence of any commercial or financial relationships that could be construed as a potential conflict of interest.

## Publisher’s Note

All claims expressed in this article are solely those of the authors and do not necessarily represent those of their affiliated organizations, or those of the publisher, the editors and the reviewers. Any product that may be evaluated in this article, or claim that may be made by its manufacturer, is not guaranteed or endorsed by the publisher.

## Footnotes

1 Henceforth we will only work with stipulative definitions.

2 We are here following Kluger et al. (2013) in treating “sensation” and “perception” as synonymous. Some argue that we ought to make a distinction between them, see e.g., (Smith, 2002; Burge, 2010; Steele, 2021) for various suggestions on how to draw that distinction. Unfortunately, though Steele, Burge and Smith all agree that we ought to make that distinction, they disagree on how to draw it. For the purpose of this paper, we will not take a stance on this issue. The framework provided in this paper ought to be easy to expand upon with a distinction between perception and sensation if required.

3 Some researchers have highlighted the distinction between active and passive fatigue, to separate between sensation of fatigue or fatiguability caused by intense work or by boredom. Passive fatigue is caused by prolonged, monotonous, boring work, whereas active fatigue is cause by prolonged task related work (Pattyn et al., 2018). This distinction is not possible without invoking an explanation (cause) or a theory dependent understanding of the specific terms in the definiens (boring, motivation). It is, however, possible for researchers interested in studying this to just add the causal part after any definition, e.g., passive fatigability is fatigability due to boredom and active fatigability is fatigability due to task related activity.

## Glossary

**TABLE 2:**

Terms	Definition
Stipulative definition	A sentence that introduces a new term and standardizes and regulates how that particular term is to be used.
Real definition	When we relate a species, the basic/smaller units of classification, to a genus, the higher order/group unit of classification.
Definiendum	The term defined in a definition.
Definiens (definientia)	The sentence or phrase that defines the definiendum.
Desideratum (Desiderata)	Something that is considered desirable or favorable.
Explanandum	A phenomenon (term or a sentence) explained in an explanation.
Explanans	The sentence that explains the explanandum.
Primitive	An undefined term that cannot be defined further and typically gets its meaning from ostensive procedures.
Connective	Words or phrases that connects two or more sentences, clauses or phrases.
Conjunction (*connective*)	“And.” The sentence “A and B” is true if A is true and B is true, otherwise the sentence is false.
Disjunction *(connective)*	“Or.” The sentence “A or B” is true if A is true or B is true or both A and B is true, otherwise the sentence is false.
Biconditional *(connective)*	“If and only if.” The sentence “If and only if A then B” is true, if A and B are true or false at the same time otherwise the sentence is false.
Ostensive procedure	Introducing the meaning of something by pointing out or showing.
